# Cytokines as Biomarkers in Rheumatoid Arthritis

**DOI:** 10.1155/2014/545493

**Published:** 2014-03-09

**Authors:** Agata Burska, Marjorie Boissinot, Frederique Ponchel

**Affiliations:** ^1^Leeds Institute of Rheumatic and Musculoskeletal Medicine, The University of Leeds, Leeds, UK; ^2^Leeds Institute of Cancer and Pathology Research, The University of Leeds, Leeds, UK; ^3^NIHR Leeds Musculoskeletal Biomedical Research Unit, The Leeds Trust Teaching Hospital, Leeds, UK; ^4^Leeds Institute of Rheumatic and Musculoskeletal Medicine, Translational Research in Immune Mediated Inflammatory Diseases Group, Clinical Sciences Building, St. James's University Hospital, Leeds LS9 7TF, UK

## Abstract

RA is a complex disease that develops as a series of events often referred to as disease continuum. RA would benefit from novel biomarker development for diagnosis where new biomarkers are still needed (even if progresses have been made with the inclusion of ACPA into the ACR/EULAR 2010 diagnostic criteria) and for prognostic notably in at risk of evolution patients with autoantibody-positive arthralgia. Risk biomarkers for rapid evolution or cardiovascular complications are also highly desirable. Monitoring biomarkers would be useful in predicting relapse. Finally, predictive biomarkers for therapy outcome would allow tailoring therapy to the individual. Increasing numbers of cytokines have been involved in RA pathology. Many have the potential as biomarkers in RA especially as their clinical utility is already established in other diseases and could be easily transferable to rheumatology. We will review the current knowledge's relation to cytokine used as biomarker in RA. However, given the complexity and heterogeneous nature of RA, it is unlikely that a single cytokine may provide sufficient discrimination; therefore multiple biomarker signatures may represent more realistic approach for the future of personalised medicine in RA.

## 1. Biomarker Research

### 1.1. General Features of Biomarkers

Biomarkers are defined as anatomical, physiological, biochemical, molecular parameters or imaging features that can be used to refine diagnosis, measure the progress of diseases, or predict and monitor the effects of treatment. They can also be associated with the severity of specific disease states.

Biomarkers can be detected and measured by a variety of methods including physical examination, laboratory assays, and medical imaging. Some biomarkers arepresent in particular groups of patients but not others, and as a result they are defined as* qualitative biomarkers* in contrast to* quantitative biomarkers* that are present at various degrees/levels in all patients. The accessibility of a biological biomarker, which is defined by the methods that are used to access the biomaterial necessary to measure it, is an important factor in relation to its adoption in clinical practice. If a biomarker can be obtained in a minimally invasive manner (typically from blood, saliva, or urine) or use tissue imaging as opposed to tissue sampling (biopsy), it will obviously be more attractive.

In the context of rheumatic diseases, typical biological biomarkers could encompass genetic markers, products of gene expression, autoantibodies, cytokine/growth factors, acute phase reactants, tissue abnormalities visualized by immunohistochemistry in synovial biopsy, a product of tissue degradation, or a cell subset that can be phenotyped and enumerated. The sources of these biomarkers could be the serum/plasma, urine, synovial fluid, tissue biopsy, or cells from blood, fluid, lymph node, or tissue. In contrast, a clinical biomarker (i.e., clinical surrogate) would constitute a physical variable (sign or symptom), a clinical judgment, or an outcome measurement that emerges as a sequel of the underlying disease process. In rheumatology, this variable may be not only joint counts, global assessment, pain score, duration of morning stiffness, and other clinical variables but also composite indices or functional, radiographic scores.

### 1.2. Specificity and Sensitivity

*Sensitivity* and* specificity* are statistical measures of the performance of biomarker using a binary classification test. This measures use used a categorical classification of patients with respect to true and false positive/negative results.

Sensitivity relates to the biomarker's ability to identify positive results. It measures the proportion of individuals which are correctly identified by the biomarker. Sensitivity is different from positive predictive value (PPV, also called precision), representing the proportion of actual positives in the population being tested.

On the other hand, specificity relates to the ability of the test to identify negative results. It measures the proportion of people without the biomarker that are correctly not assigned to the condition. Sensitivity may be affected in case of a number of indeterminate test results. It is possible to exclude these cases from analysis or, alternatively, to treat them as false negatives (which gives the worst-case value for sensitivity but also underestimates it), but such exclusions should be stated when quoting sensitivity.

An optimal biomarker would aim to achieve 100% sensitivity (i.e., predict all people with the condition) and 100% specificity (i.e., not predict anyone from the control group). For any biomarker, there is usually a trade-off between the measures and their impact, setting acceptable limits and allowing detection of false positive (lowering specificity), but limiting false negative (increasing sensitivity).

Taking the example of anticitrullinated peptide antibodies (ACPA) in RA, sensitivity is usually reported around 68% and specificity is reported at 95% [1]. However, sensitivity is highly dependent on the group of individuals tested and values observed in established diseases that do not reflect the general RA patients' population or early disease. Indeed, in patient with recent onset of symptoms, studies have shown that sensitivity is much lower (ranging from 35% to 50%) even if specificity remains closer to 95% [2].

Multivariate markers are as follows: the concept of biomarker algorithm or multivariate biomarkers has recently been developed based on the observation that a single biomarker is often insufficient to predict the outcome of interest, when a combination of biomarkers is better at achieving the prediction. It is usually observed that multivariate biomarkers perform better in replicate studies than univariate biomarkers.

### 1.3. Need for Biomarkers in Rheumatoid Arthritis (RA)

RA is a complex disease that develops as a series of events often referred to as disease continuum. Research into the preclinical and early phases of RA recently reviewed these events and categorised groups of individuals based on risk factors [3]. According to this new terminology, healthy individuals without RA are described as having potentially two main types of risks: (i) a genetic risk, for example, if they carry the shared epitope allele and (ii) an environmental risk if they smoke. They, however, do not present any laboratory evidence of symptoms or any signs of inflammatory arthritis. The first phase of RA disease progression would then be a state in which individuals develop features of systemic autoimmunity that can be measured by laboratory investigations and are known to be associated with RA (such as ACPA) [3] and more recently with carbamylated protein [4, 5]. These individuals still do not present any symptoms or signs of inflammatory arthritis. A further stage is then defined by the appearance of symptoms (such as arthralgia/morning stiffness), still with no evidence of any clinical synovitis. These individuals can come from both the genetic and environmental risk groups, from the systemic immunity group, or from the general healthy population. Finally, the last progression stage is represented by the development of clinically apparent inflammatory arthritis that may not yet fulfil the criteria for RA diagnosis [6], and hence it is being termed undifferentiated arthritis but is likely to evolve towards RA.

There are many situations in RA, which would benefit from biomarker discovery, considering that biomarkers may be broadly classified as diagnostic (detected when disease is present), prognostic (associated with disease outcome), or predictive markers (associated with drug response). Diagnosis is obviously an area where new biomarkers are still essential as RA is a condition where diagnosis relies on signs and symptoms even if recent progress has been made with the inclusion of ACPA to the recently updated criteria [6]. However, in RA diagnosis, the performance of biomarkers may greatly depend on the duration of symptoms at the time of test, the current level of inflammation, and the amount of destructive processes already undergone, as well as on the type of tissue tested. Prognostic biomarkers which predict the future course of the disease and provide information regarding the outcome irrespective of therapy would be very important in foreseeing the evolution of undifferentiated arthritis towards RA or with respect to the severity of RA which can be quite variable. Prognostic biomarker validation is therefore relatively straightforward, as it is associated with the disease and the patient and can be established (at least in theory) using data from a series of patients treated with standard treatment. The discovery of specific biomarkers for poor prognosis would, for example, enable early intervention and intensive treatment. Risk biomarkers for predicting rapid evolution or cardiovascular complications, for example, remain highly desirable. Monitoring biomarkers would be useful in predicting relapse and candidates are available using flow cytometry based cell subset phenotyping [7–9]. Predictive biomarkers would separate an RA patients' population with respect to their outcome in response to a particular event taking place (i.e., particular therapy). They are therefore present/absent prior to the outcome occurring and have obvious applications with the greatest potential to affect clinical practice by targeting drugs to relevant patient subgroups. Biomarkers allowing the selection of an optimal drug for a particular patient (acknowledging that certain subset of patients respond better to certain drug than others) may represent another essential step in patients screening that would notably allow personalised medicine models to be developed, tailoring therapy to the individual, shortening time from onset to effective treatment, improving cost and risk-benefit ratios of drugs, and ultimately achieving high response rate with minimal toxicity [10]; however, in patients with long-standing RA heterogeneity in disease presentation, there remains a major obstacle even when using biomaterial as close to the disease site as synovial tissue [11].

There are several sources of tissue and body fluid that can be considered for biomarker discovery programs in RA. The suitability between the levels of invasiveness and the benefit provided by the biomarkers is however to be considered as well as the level of investigation patients would be likely to accept. Diagnostic biomarkers, considering the prevalence of the disease (1-2%), would need to use biological material which is easily accessible and a method of collection which would not impact on the progression of the disease. Blood and urine therefore appear more suitable compared to synovial tissue or fluid particularly at this early stage of the disease where mostly small joints are involved. Later in the disease continuum, tolerability for more invasive procedure such as fluid aspiration or biopsy collection would provide material reflecting the disease site more closely allowing for individual variability to be taken into account for a personalised medicine approach.

## 2. Cytokines as Biomarkers 

### 2.1. Cytokine Classification

Cytokines are small proteins which play important roles in cell signalling. They are secreted by a variety of cellular sources acting either on the cell producing them (autocrine) or on the surrounding cells (paracrine). They are classified as proteins and sometimes peptides and can also be glycosylated. Cytokines usually circulate in very small amounts (picomolar 10^−12 ^M) and, nonetheless, their concentration can increase up to 1,000-fold when required. Cytokines have originally been identified in the context of the immune system; however, it has now been shown that they are produced by and influence the behaviour of a variety of nonimmune cells. Cytokines are often referred to as “growth factors” by association with one of their most common effects, the induction of cell proliferation, despite a wide spectrum of roles in survival, apoptosis, differentiation, and functional activation (contribution to the immune response).

Over the years, cytokines have been categorized into various classes, families, or superfamilies. It has been done using either their numerical order of discovery (notably, in the interleukin family, currently up to IL-38) or a given functional activity (e.g., the larger tumour necrosis factor family). In that case, they are further divided between cytokines which enhance cellular immune responses (type 1) as opposed to those which favour antibody responses (type 2). This subclassification is performed using their function (early or late, innate or adaptive, pro- or anti-inflammatory, mitogenic, regulatory, survival functions) or, sometimes, using their primary cell of origin (monokine, lymphokine). More recently, classification has been achieved using structural homologies shared between related molecules. Nevertheless, despite sharing sequence homology and some promiscuity between their receptor systems, cytokines demonstrate specificity in their function and even opposing functions within members of the same family (best illustrated in the TNF superfamily).

Methods of detection for cytokines also vary considerably. Enzyme-linked immunosorbent assays (ELISAs) have long been considered the “gold standard,” but, nowadays, the development of multiplexing technology has allowed biomarker programs to investigate whole cytokine networks as opposed to individual candidates notably enabling large data sets to be generated from small body fluid volumes. Several multiplexing technologies are now available, including the bead-based immunoassay (often referred to as Luminex assay), membrane-based ELISAs, and Mosaic ELISAs, as well as cytometric bead arrays (CBAs). Concerns have been raised related to the sensitivity of some multiplex solid-phase assays [12] as well as interference from heterophilic antibodies [12–19]. This is of particular relevance in autoimmune disease where rheumatoid factor (RF), a heterophilic autoantibody directed against the Fc portion of IgG is present notably in RA [12, 20–25].

### 2.2. Variability and Limitations of Cytokine Measurements

#### 2.2.1. Patient Related Variability

There are a number of features and conditions that can influence cytokine production which are related to donor variability in both health and disease. Some of these characteristics are unlikely to change during treatment (genetic/ethnic background, gender, and age); however, others may greatly limit the ability to use cytokines as biomarkers in everyday practice. These factors such as diurnal rhythmicity and sample handling factors (collection methods, storage, and plasma versus serum) may influence the measurement of cytokines and are also likely to change with not only therapy but also stress and cachexia. Such factors are likely to contribute considerably to the disparities seen among similar types of clinical studies [53–55].

*(1) Age and Gender Effects.* Comprehensive analysis of 30 different biomarkers in *≈*400 healthy donors, ranging in age from 40 to 80 years, showed an increase in serum interferon-inducible chemokines (MIG and IP-10), eotaxin, and soluble TNFR-II with advancing age [56]. Multiple studies discussed differences in cytokine production associated with donor age, and several reports have demonstrated that chronic, low-grade inflammation is linked with the aging process [57–59]. An age-related increase in IL-6 concentration has been reported in serum, plasma, and supernatants of mononuclear cell cultures obtained from elderly subjects [60, 61]. Some studies demonstrated that plasma levels of tumor necrosis factor (TNF) are elevated in elderly populations [59, 62–64]. Conversely, other cytokines regulating T cell functions, such as IL-2, may be decreased with aging. The suppressed production of IL-2 leads to a small clonal expansion of T cells thus decreasing the ability to develop specific immune responses [61]. Modifications of the immune system are globally evaluated as a form of deterioration called immunosenescence. However, ageing is also accompanied by a chronic low-grade inflammation state, showed by a 2 to 4-fold increase in serum levels of inflammatory mediators which act as predictors of mortality independently of preexisting morbidity. This proinflammatory status underlies biological mechanisms responsible for decline in physical function, and inflammatory age-related diseases are initiated or worsened by systemic inflammation [65]. The term “inflammaging” has been coined to explain the underlying changes common to the most age-associated conditions [66, 67].

Longitudinal cytokine production in paediatric and adult patients identified multiple differences in terms of proinflammatory cytokines such as IL-6, IL-8, IL-1alpha, IL-1beta, MCP-1, MIP-1alpha, IL-15, IL-5, IL-17, IL-18, and IP-10 and of anti-inflammatory cytokines such as IL-10, G-CSF, IL-13, IFN-gamma, and IL-4 between the two groups [68]. Altogether, the age of onset in RA patients is to be taken into consideration as it may reflect the cytokine production profile. Men and women also present with gender related differences in the way their immune system responds to challenge [69]. Females demonstrate better B cell-mediated immunity than age-matched males (with higher immunoglobulin levels, stronger antibody responses, and increased resistance to certain infections). Gender also influences T cell immunity, females having greater resistance to induced tolerance, an increased risk to reject grafts, and higher levels of IL-1, IL-4, and IFN-gamma in contrast to men who produce more IL-2, -4, and -13 and whose monocytes secrete more IL-1beta and TNF-alpha [70]. Differences in cytokine production profile have also been suggested to play an important role in the gender bias with regards to the ratio of relapsing remitting and secondary progressive multiple sclerosis [71] as well as susceptibility to urinary infection [72]. Aging has also been associated with alterations of the musculoskeletal system and a decline in sex hormone levels, which have a central role in the regulation of bone turnover. The effect of age combined with gender on cytokines and markers of bone metabolism production showed an increased proportion of T cells producing IFN-gamma and IL-2, IL-4, IL-10, and IL-13 particularly in elderly women after menopause [73].

*(2) Circadian Rhythm.* Cytokines present a circadian pattern. For example, IFN-gamma, TNF-alpha, IL-1, and IL-12 production exhibits distinct diurnal rhythms that peak in the early morning [74] and are related to the rhythm of plasma cortisol and melatonin [75–77]. Taking IL-6 as an example, notably with respect to RA, IL-6 demonstrates important variation in serum or plasma levels in healthy subjects over a day period with a particular biphasic rhythm [78] altogether amounting up to a CV >23%. After correction for analytical variation, a rise in serum IL-6 in the late evening and the early morning has been reported in RA [78–82] as well as high variations between and within days not necessarily indicating rhythmicity [54]. Therefore, only IL-6 changes over twice the biological variation (>50% difference) should be considered significant [78]; however, in order to obtain comparable and meaningful results, the time of sample collection should be synchronized, with a morning sample collection time being ideal. This does not affect all cytokines but is not particularly well described for many and should be considered if/when validating a biomarker for clinical use.

*(3) Food Intake.* Long-term food intake patterns (i.e., obesity or weight loss) have been shown to affect circulating cytokine levels, notably TNF-alpha [83]. Postprandial cytokine levels are also affected by feeding; notably circulating IL-6 levels are increased, while TNF-alpha levels are decreased [84–87]. Food supplements (in particular, antioxidants such as glutathione and vitamins E and C) can attenuate the feeding-induced rise in plasma cytokines [88, 89]. Hence, patients should be instructed to maintain normal dietary habits and avoid food supplements prior to sample collection if the cytokine of interest is sensitive to such regulation [90, 91].

*(4) Exercise.* Physical exercise can affect cytokine levels in the circulation [54, 92]. While plasma cytokines are produced by many cell types, muscle cells are a major source of secreted cytokines during exercise [93, 94]. However, these particular responses are highly specific to the exercise protocol and physiological strain (duration, nature of the exercise, and intensity) [95, 96]. Several studies reported elevation of plasma IL-6 in healthy subjects, which peaked at the end of exercise. The magnitude of the IL-6 response was related to the duration and intensity of the muscle work, the mass of muscle recruited, and the subject's endurance capacity [78, 97–99]. In patients with RA, no changes in serum IL-6 were found after cycling. This could be due to the less strenuous exercise performed by the RA patients because of their widespread joint pain [78, 100]. In contrast, evidence suggests that the prophylactic effect of prolonged, endurance type exercise protocols may be mediated via the induction of an anti-inflammatory environment (increases in circulating levels of IL-1RA and IL-10) [101]; however, how/whether both are linked remains poorly defined. There is nevertheless consensus that exercise training protects against some types of cancers by enhancing antitumour immunity and reducing inflammatory mediators. Altogether, any unconventional strenuous activity prior to blood collection for cytokine measurements should be avoided.

*(5) Stress.* Stress and emotional problems were also shown to influence cytokines levels; however, studies yielded contradictory data with decrease, increase, and no change in proinflammatory cytokine production being reported [102–104]. Nevertheless, lower self-rated health was associated with higher levels of inflammatory cytokines IL-1 and TNF-alpha (controlling for age, education, and physical health) [104].

#### 2.2.2. Preanalytical Related Variability

There are several specific problems posed by sampling conditions (i.e., preanalytical issues) in addition to those described above. Cytokines act either in a paracrine or an autocrine manner as they are released and consumed locally, close to the site where the immune reaction occurs. Therefore, they are rarely detectable in peripheral blood and then only at low levels [105]. Blood may thus only partly reflect pathologies, including RA, and therefore not be the material of choice. The half-life of many cytokines is also measured in minutes; hence, the time lapse between the collection and processing of the samples may be a significant factor limiting the use of cytokines as biomarkers.

Data reproducibility can be affected by normal human variability, which is relatively easy to control in model systems (i.e., in cell culture or even animal models) but is much harder to control in real subjects. Designing and testing the sample collection (i.e., anticoagulants, stabilizing agents) and handling (temperature, elapsed time from collection to initial processing, and endogenous degrading properties of the analyte) and processing protocol/method will represent key elements in the successful development of any biomarkers [106].

*(1) Serum or Plasma?* In body fluids, cytokines can exist under multiple molecular forms related to posttranslational modifications (i.e., glycosylation), monomers/polymers, precursors, and degradation products or complexed with other proteins [107]. Such molecular forms can behave differently in assays used to determine their levels; therefore, choice of different analytical techniques will be determinant in selecting blood preparation. Serum and plasma are not interchangeable, and the use of one or the other will determine which technique should be used for analyte quantification (see Table 1). Therefore, a lack of consensus exists with respect to the optimal type of specimen to measure cytokines, and the question remains open as to whether plasma or serum should be used. It is important to determine if the method used to collect and prepare the sample may introduce alterations to the cytokine to be tested (i.e., cytokines, either individually or on all proteins in the sample) or whether certain preparation methods are desirable or not for certain cytokines [108].

*Serum* represents the soluble fraction of clotted blood. Serum preparation involves the removal of fibrinogen, platelets, and other circulating proteins. Clotting takes a minimum of 30 minutes but no longer than 60 minutes. Blood should then be centrifuged for 10 minutes and serum should be separated from the clot. Blood cells may get activated during the clot formation and cytokines may be released as a result (such as IL-1, IL-6, and CXCL8) [27, 90, 109, 110]. Rapid sample processing is therefore essential to accurately measure cytokines due to platelet release (i.e., IL-1, IL-6, sCD40L, and others) [21]. For this reason, in order to have correct estimates of specific cytokine levels, it may be preferable to measure them in plasma rather than in serum [34, 111]. This notably raised issues when comparing serum and plasma levels for TGF, IL-1, IL-6, IL-7, and so forth [38].

*Plasma* is the soluble fraction of anticoagulated blood. To obtain plasma, various anticoagulants can be used (ethylenediaminetetraacetic acid (EDTA), lithium/sodium heparin, and sodium citrate). Cytokine measurements were shown to be affected by the anticoagulant used [78] and, notably, lithium heparin and sodium citrate were shown to affect levels of IL-6 and TNF-alpha compared to EDTA plasma [35, 112, 113]. Citrate plasma collection also results in the reduction of total protein concentration due to the volume of citrate anticoagulant diluting the blood, in addition to an osmotic withdrawal of water from blood cells [114]. Endotoxin present in lithium heparin tubes when sterility is broken [113] can also induce cytokine release from cells, whereas EDTA inhibits endotoxin [26, 31]. Variation in cytokine levels could be attributed to anticoagulant-induced release of cytokines by blood cells notably in heparin plasma but not in EDTA plasma, [115]. Altogether, plasma collection with use of EDTA seems to bring the most consistent results [34, 35, 116] and more closely resembles data obtained in serum [31, 35, 39, 78, 90, 117]. Cytokine stability also appears increased in EDTA plasma [26, 118] perhaps through EDTA's role as a protease inhibitor. Further mechanisms can explain differences in stability such as change in degradation rate or modification of cytokine's structure due to the differential presence of other proteins in EDTA plasma compared to citrate plasma or serum (i.e., soluble forms of receptors) leading to a lack of recognition of the antibodies used in the ELISA. The limitation in using plasma remains the need for rapid separation after collection with changes occurring as soon as 30 minutes after sample collection [34].

Over the recent years, improvements in the collection tubes have been made, notably with the use of serum separator tubes, which include a gel that serves as a barrier between serum and the clot [106], or the substitution of plastic for glass allowing direct centrifugation [119].

Altogether, no single type of sample is optimal for every analyte; therefore, the development of assays for individual cytokines should require optimisation on a case-by-case basis, although it would be recommended to collect both serum and EDTA plasma.

*(2) Time to Processing.* Time is an important factor that needs to be accounted for when measuring circulating cytokines which have a relatively short half-life and an important risk of degradation notably when comparing them to other proteins such as antibodies [26, 34, 120]. Changes in the amount of cytokine detected depend on the delay and duration of sample processing and are likely due to altered production by cells after blood collection [31, 54, 120], or their binding by other proteins (i.e., soluble receptors or cells surface receptor) [42, 120, 121], or, finally, due to enzymatic activities (proteases) leading to cytokine digestion (see also Table 1). Rapid processing of samples is therefore essential, notably as samples obtained from patients often present with higher concentrations or increased activity of proteases or other factors which render specimens even more unstable than those obtained from healthy controls [111]. Ideally, samples destined for cytokine detection should be collected in sterile (endotoxin-free) tubes and processed quickly with a minimum of 30 minutes of clotting time but no longer than 60 minutes after blood draw, independently of the type of tube used (plasma or serum). Processed plasma or serum should be frozen at −80°C as soon as possible in small aliquots to avoid repeated freeze-thaw cycles [107, 122]. Some reports proposed to keep samples refrigerated at 4–8°C (but not on ice) after clotting for the duration of processing as room temperature favours proinflammatory cytokine degradation such as IL-6 but conversely stabilises TNF-alpha [26, 34, 120, 123, 124]. Most cytokines are relatively stable with the well-known exception of TNF-alpha and IL-6 [42, 125, 126]; therefore, the interval between blood draw and separation should not exceed 3–24 hours, even when the tubes are stored at 4–8°C and only when EDTA tubes are used (TNF-alpha however cannot be reliably measured any longer), although many cytokines have not been sufficiently tested [26, 35, 37, 78].

The effects of centrifugation speed are more difficult to evaluate. Gradual increase in* g* values (from 200 to 13,000 g) is necessary to achieve graded depletion of platelets and leucocytes from plasma; however, it reduces the levels of certain cytokines (i.e., sCD40L) [52]. Of note, the use of blood tubes with gel separator imposes a certain centrifugation speed to allow separation of serum and cells but does not allow tubes to be chilled before or during centrifugation [127].

*(3) Storage Temperature and Freeze-Thaw Cycles.* By and large, most cytokines and soluble markers are quite stable if frozen (see also Table 1). Storage conditions, however, vary with a choice of temperatures from short-term storage at room temperature (RT) or 4–8°C (days) to medium term (a few months) more often between −20/−30°C and long term (years) at −70°C. Direct comparison of several cytokines in plasma stored for 20 days at RT, 4°C or −70°C, showed remarkably stable levels (IL-10) except for TNF-alpha particularly at room temperature [128]. In contrast, a more recent study of reliability and reproducibility of cytokine measurements in healthy donors [122] showed that, while most cytokine measurements are stable for up to 2 or 3 years when stored at −80°C (see details in Table 1), they do not all remain stable after repeated freeze-thaw cycles. After 4 years, most cytokines were degraded. Importantly in RA, levels of certain cytokines such as TNF-alpha increase with each successive freeze-thaw cycle [54, 90, 122]. Therefore, it remained difficult to compare studies from different centres even when using the same assay for cytokine measurements (i.e., commercial kit) [39]. Altogether, the consensus would recommend storing specimens at −80°C in as many small aliquots as possible to limit freeze-thaw cycles [129].

#### 2.2.3. Analytical Variability

*(1) Assay Type.* Numerous immunoassays exist to measure cytokines both in their protein form: ELISA, nitrocellulose, or other solid phase assays, immunohistochemistry, and bead-based flow cytometry multiplex immunoassays, and in their molecular form: reverse transcriptase PCR, microarrays, and* in situ* hybridisation (Table 2). Immunoassays use antibody to immobilise cytokines on a solid surface and then identify them with different methods for quantification using colorimetric enzymatic reactions, fluorescence, luminescence, or even, in the past, radioactivity. There are two types of assays using either one or two antibodies: one being for cytokine capture adding more specificity compared to total protein plastic binding and the second one being for detection. The major benefit to using antibodies is that assays are more specific and reproducible. Several platforms for the detection and quantification of cytokines exist. There is no universal best method for cytokine measurements; however, the oldest technique (ELISA) is often used as gold standard despite the fact that direct comparison between many commercially available kits has not been performed. Cytokines show complex protein structures (monomers/polymers, precursors, various degrees of glycosylation, and degradation products) and their activity often depends on the integrity of such structure. Minor changes that may not be detected by physicochemical measurements, immunoassays, or biophysical methods may have dramatic effects on biological activity (e.g., cytokines may lose most of their biological activity but will remain detectable if measured as mass) [130]. The presence of soluble forms of the cytokine receptors (i.e., sIL-2R, sIL-7R, and sTNF-R) in biological samples and the existence of autoantibodies to cytokines (i.e., anti-TNF-alpha, IL-6, and IL-1) [131] may or may not interfere with the recognition of cytokines by either capture or detection of antibodies [39, 132–134]. Each method has advantages and limitations and should be carefully selected with respect to the research purpose. To date, most cytokine measurements in large studies essentially used ELISA, which is widely accepted as the “gold standard” method. The main limitation of ELISA remains that it allows the characterization of a single cytokine at a time, hence the development of multiplex technologies. One of the most commonly used methods for this is the multiple target based assay [135], which can measure up to 100 different analytes per sample from a small volume of body fluid [136], or more recently the cytometry bead assay (CBA) which relies on bead as solid phase and uses flow cytometry to discriminate between analytes [137]. Multiplex measurement of inflammatory cytokines in human serum by electrochemiluminescence assay was recently developed [138]. These multiplex assays are in concept close to ELISAs and dependent upon the careful choice of the capture/detection antibody pairs and proper buffering to minimize differences in assay performances [135].

Several studies have compared cytokine levels determined by ELISA and multiplex immunoassays with results showing either good or poor correlations between the methods. Therefore, it is not surprising that discrepancies in data comparing measurements of cytokines were observed when different commercial/manufacturers' kits were used, even if preanalytical conditions of samples collection, separation, and storage were identical [85, 136, 139]. The use of different antibody clones to capture and detect cytokines is also likely to affect results and change the level of sensitivity of such assays. Furthermore, some monoclonal antibodies recognise different molecular complexes (monomers/polymers, precursors, glycosylation, degradation products, or total bioactive or inactive forms) [140]. In summary, comparison of the same samples (eliminating preanalytical bias) using several commercial ELISAs demonstrated that variability was mostly attributable to each assay (measuring TNF-alpha, IL-1 alpha and IL-1beta, IL-6, IL-2, IFN-gamma, and the soluble receptors of IL-2 and TNF) but yielded comparable results when the same ELISA was used at different centres [85, 139]. The nature of the different pairs of monoclonal antibodies employed in each ELISA is most likely the major source of variability, but these findings also highlight the necessity of establishing international standards for all immunoassays as ranges are also widely variable between these commercial assays. If cytokines are to be employed as clinical biomarkers for diagnosis, prognosis, and prediction, accurate and reproducible assays need to be adopted internationally.

*(2) Interferences.* Interferences within immunoassays are numerous, complex, and usually difficult to resolve. Proteins can show an altered expression pattern in more than one disease. The presence of lipids, complement factors, and other complex molecules in the blood was also shown to interfere with a number of assays. Human anti-animal antibodies present in biological samples (especially human anti-mouse antibodies) may cause problems; however, these may be blocked by the use of multiple species serums as blocking agents [141]. Haemolysis interference occurs rarely; however, it can affect some analytes. Lipaemia interferences were confined when using immunonephelometric and immunoturbidimetric assays, and, ideally, grossly lipaemic samples should be cleared (using ultracentrifugation of lipaemic samples with correction for volume displacement errors) or discarded. Antigen excess may, in some cases, result in false low values [142]. Complement factors and paraproteins are capable of binding to assay antibodies (capture and detection) causing interferences [142]. In addition, biological fluids may also contain naturally occurring antibodies to a variety of proteins, including cytokines themselves. Such antibodies, although at variable levels notably between normal donor and patient populations, can interfere with assays particularly if they share the same epitope on the cytokine [143]. The existence of autoantibodies against cytokines has been documented for TNF, IL-1 (alpha and beta), IL-2, IL-6, IL-8, IL-10, and IL-18 [144–148]. Autoantibodies against IL-1 are the best studied. Their prevalence is high with an affinity which can reach up to 10^−11^ M that is very similar to the affinity of antibodies developed for immunoassays [140]. However, the main issue remains heterophilic antibodies. These antibodies are naturally produced polyclonal autoantibodies with low specificity directed against multiple poorly defined antigenic immunogens. Most often, they are present in individuals exposed to foreign proteins (e.g., domestic animals and household pets). The occurrence of false positives in immunoassays [13–16] is often the result of heterophilic antibodies nonspecifically bridging the assay antibodies [18, 19]. As a result, studies have often overestimated cytokine levels notably when using the Luminex technology [12].

Blood samples from patients with autoimmune diseases, such as RA, may be problematic due to the presence of additional disease related autoantibodies [149]. RF is an autoantibody directed against the Fc portion of IgG and is found in 75% of patients presenting with RA as well as other diseases such as Sjögren's syndrome, infective endocarditis, systemic sclerosis, and systemic lupus erythematous (SLE) [24]. RF was shown to exhibit most of the heterophilic antibody properties with several antigen cross-reactions [25] and hence immunoassay in RA is particularly sensitive to this issue and needs careful evaluation for RF interference [12, 150–153]. Heterophilic immunoglobulin may further develop as a result of treatment with drugs attached to mouse (or humanised) monoclonal antibodies.

Several methods for removing heterophilic antibody (notably RF) from patients sera have been developed [21, 154–156]: (i) initial serial dilutions may be recommended, particularly when results demonstrate nonlinearity suggesting the presence of heterophilic antibodies, (ii) the use of blocking reagents such as nonimmune serum from the same species as the assay antibodies, species-specific polyclonal IgG, and multispecies mixture (20% normal mouse serum, 10% goat serum, and 10% rabbit serum), as well as commercial reagents such as HeteroBlock [155], and (iii) the specific removal of immunoglobulin G using sepharose-L or polyethylene glycol precipitation (PEG 6000) has also been used. These methods act by physical removal of the immunocomplexes [155], which are then separated by centrifugation. Several reports have been published investigating interference by heterophilic antibodies in RA sera using solid phase multiplexing technology including Luminex [23, 155, 157, 158], a glass chip/chemiluminescence platform, or a multiplex sandwich ELISA. They showed clear interference (i.e., false positive) in RF-positive sera but not in negative samples [157]. In our lab, all methods were efficient at blocking/removing relatively low RF quantities in serum samples from RA patients [12]; however, none of these methods were effective when high levels of RF were present (>100 U/L) and residual RF still generated false positive results particularly when using certain types of assays (Luminex) but not others (ELISA, membrane-based ELISA, Mosaic ELISA, or CBA).

*(3) Standardisation and Quality Control.* Commercially available immunoassays in the form of “kits” are now extensively used. Considerable variability can arise from the use of these assays. Differences in measured levels of cytokines in identical samples using different standards ranged from 10- to 100-fold [130, 159–161]. Some issues are related to the use of different epitope specificity of the antibodies, while others arise due to the use of various reference preparations (standards) for calibrating the assays [55]. Comparison of cytokine levels requires unit definition by a standard that is assay independent, which, once defined, should be used by any laboratory, thus providing a means of ensuring uniformity worldwide [130]. Variations as a result of differences in standards account for as much variability as sample collection, processing, or storage issues [31, 42, 125, 159–168]. All cytokine assays should therefore be calibrated against such standards, regardless of assurances provided by the kit manufacturers. Notably, results of cytokine assays should be reported in picograms or nanograms per milliliter instead of arbitrary units. Major international efforts to organise standardisation of cytokine measurements have been conducted by the World Health Organisation, (see details at http://www.nibsc.ac.uk/products/biological_reference_materials.aspx), The National Institute for Biological Standards and Control (NIBSC), and the Biologics Evaluation and Research (The National Institutes of Health (NIH), Bethesda, MD 20205, USA) (http://www.who.int/biologicals/) [130, 131, 169]. Nonetheless, baseline values for a lot of cytokines have not yet been reliably established in healthy controls (despite a range suggested by most manufacturers), making it difficult to interpret the biological significance of minor variations in cytokine levels in patients [170]. Furthermore, some cytokine assays are sensitive at relatively high concentrations that may not always cover the physiological range even in diseases [12]. Quality control (QC) measure is also an essential step of biomarker development. Therefore, during the analytical phase, QC should be considered to document analytical performance during any studies to determine the acceptance or rejection of an analytical run during postanalytical sample analysis [136, 171]. QC samples could be prepared to evaluate the lower, middle, and upper performance limits of an assay. A number of validation samples (at least five different concentrations) should also be used to estimate intra- and interrun accuracy/precision and stability [136, 172, 173].

## 3. Cytokines Network in RA

Over the years, increasing numbers of cytokines have been involved in RA pathology, further to those used as target of cytokine-blocking therapies which emerged from the hypothesis that the most abundant cytokines present in the joint were more likely to be pathogenic. A large number of cytokines are detected at the disease site (through both mRNA and protein quantification) in both synovial tissue and fluid, where they have a role in perpetuating inflammation, cartilage destruction, and bone remodelling associated with RA. Several methods of detection (ELISA, immunohistochemistry) identified TNF-alpha and IL-1 as major players in the network of cytokines, notably directly expressed at the disease site in joint tissue or fluid. IL-6 and IFN-gamma are also present as well as GM-CSF and LIF. More recently, other cytokines were added to this list (IL-7, IL-15, IL-17, IL-18, IL-21, and MIP-1 notably) together with cytokines with activities targeted towards fibroblasts (TGF-betas notably) and finally several growth factors (PDGF, EGF, and VEGF) [174] and chemokines (IL-8, SDF-1, RANTES, and MCP-1). Cytokines favouring survival of infiltrating cells have also been detected (such as the pairs between IL-7 and T cell or BAFF and B cells). However, if proinflammatory cytokines (TNF-alpha, IL-1, and IL-6) are abundant in all patients, cytokines classically defined as anti-inflammatory and regulatory (IL-4, IL-10, IL-13, and TGF) [175, 176] as well as antagonist receptors (IL-1RA, or soluble IL-2R, or TNF-R) are also present. Most of these cytokines have dual roles with anti- and proinflammatory aspects depending on the context and the network they form; hence, studying their roles and actual effects is particularly complex. The redundancy and synergy between the effects of all cytokines in such an intricate network may further explain the inadequate response to single blockade therapy notably in established disease [175].

The interplay between cytokines, where excess of one may result in suppressed production of another, further complicated by interactions with soluble receptors for some of these cytokines, renders data interpretation challenging (notably for TNF-alpha and IL-1) [88, 89]. The relationship between blood and tissue is often complex and translating findings often proves difficult if not conflicting. Data on cytokine levels in humans in relation to disease activity is still limited. Increased levels of cytokines such as IL-l, IL-6, and TNF have been interpreted as an indicator of the inflammatory state. It is unlikely that these cytokines could serve as “biomarkers” in inflammatory disease, as they are linked to the disease biological processes, hence not specifically associated with a particular disease. Additionally, lack of correlation is often observed between cytokine levels (in serum/plasma) and clinical endpoints.

On the other hand, the absence of a cytokine in disease is particularly difficult to interpret. As indicated above, there may be multiple reasons for the inability to detect a cytokine when actually it is expected to be found. Even in the absence of specific or nonspecific inhibitors, excessive consumption of a cytokine versus lack of its synthesis is hard to dissociate. As an example, IL-7 levels were reported to be low in RA serum [177–179]; however, they are high in synovial fluid and tissue. The presence of high levels of sIL-7R in serum [180] may explain this discrepancy and the associated loss of biological activity [177, 181].

Despite these limitations, there are some cytokine biomarkers, which appear to be relevant in RA. IL-6, despite not being disease specific [78, 92, 182], was shown to be more sensitive than CRP (despite being directly correlated with it) for the prediction of therapeutic response of RA patients to rituximab [183]. Similarly, IL-7 was shown to have some value as diagnostic biomarker associated with potential for more erosive disease [179].

### 3.1. Differential Cytokine Expression between Diseases

Over the years, many studies provided evidence of differential expression of cytokines between healthy control (HC) and diseases such as RA, osteoarthritis (OA), ankylosing spondylitis (AS), psoriatic arthritis (PsA), reactive arthritis (ReA), systemic lupus erythematosus (SLE), or gout. These initially used functional assays measuring the production of cytokines in variable cell subsets using intracellular expression of cytokines (in CD4+ or CD8+, T cells or B cells, or monocytes), ELISA, ELISOPT, or mRNA quantification. Several important observations were derived from these experiments and the tables below summarise all this data as well as tissue sources and technology/experiment.

*In vitro* assays removed the microenvironment context; however, they reflect good the capabilities acquired through exposure to the priming effect that such microenvironment may exert (i.e., Th1/Th2 polarization, transition from naïve to memory). Altogether, they demonstrate the dysregulated expression of certain cytokines in T cells subsets notably and increased expression by monocytes in RA patients. Importantly, all cytokines tested were shown to be increased, with the exception of IL-2 and IL-4. Interestingly, RA patients' T cells showed hyporesponsiveness to stimulation of the T cell receptor (TCR) pathways and hardly produced any cytokines despite evidence of previous activation (memory phenotype) [184]. This deficit was attributed to chronic exposure to TNF-alpha [185] and/or abnormal RAP1 signalling [186–188]. The classic model of T cell naïve/memory differentiation is perturbed in RA. T cells despite being naïve with respect to antigen stimulation [189] express chemokine receptors which facilitate trafficking to sites of inflammation [7, 177]. This phenomenon was hypothesized to result from cytokine activation notably of naïve T cells (by IL-6 and TNF-alpha) bypassing the need for an antigen to achieve activation [190, 191]. Similar cells were found in RA joint (but not OA) [192] where they enable TNF-alpha production by monocytes in an antigen-independent manner. These properties of cytokine activated T cells were further extended to chemokine production and were confirmed* in vivo* using a cytokine cocktail containing IL-2, IL-6, and TNF-alpha [193]. Such increased ability to produce all types of cytokines reflects the chronic stage of the disease but nevertheless gives insight into potential candidates for further biomarker program.

### 3.2. Differential Cytokine Levels in RA Sera or SF

There are several studies comparing circulating levels of cytokine, they often show discrepancy in their results, and most do not use the appropriate biomarker development strategy. IL-1beta and TNF-alpha are increased in RA [194] and such profile is accentuated in active diseases compared to clinical remission [195]. In contrast, low levels of IL-2 and IL-7 were reported [177, 179, 194, 196]; however, those may be due to high levels of soluble sIL-2R and sIL-7R which are also present. IL-6 could not be detected in HC serum, while serum IL-6 levels are substantially increased in RA with significant circadian variations corresponding to the circadian rhythm of symptoms in RA [79]. High IL-7 [197] and IL-16 [198] were detected in sera and SF of RA patients compared to OA and are also confirmed in synovial tissues by mRNA levels. Certain cytokine levels were related to disease parameters such as IL-1RA and the number of tender and swollen joints [199], IL-18 (both sera and SF) and disease activity [200, 201], and IL-7 in the tissue (both mRNA and protein) with local levels of inflammation measured during arthroscopy [196]. IL-21 is highly produced in the synovial fluid of RA patients compared to paired serum specimens as well as healthy control sera. The increased levels of IL-21 correlate with those of IL-17 [202] and an association between levels of IL-21 and Th17 cells responses in the RA synovium was shown [202].

Similar increased serum levels of many cytokines were indeed found in other rheumatic diseases: notably PSA [203–205], SLE [206, 207], AS [208–210], and scleroderma [211, 212] (IL-1, IL-6, IL-7, IL-8, IL-10, IL-12, IL-16, IL-17, IL-18, and IFN-gamma, TGF-beta, or TNF-alpha, as well as IL-1RA and sIL-2R or leptin) suggesting that such rises may reflect inflammation rather than being disease specific. Therefore, the biomarker value of either one of the cytokines, or a combination of them, will likely depend on whether their disease specificity can be verified.

### 3.3. Cytokines as Diagnostic Biomarkers for RA

The early diagnosis of RA is critical, as it has been demonstrated that a therapeutic window of opportunity is available very early in the development of RA, when disease can be stopped efficiently, preventing structural and functional damage and leading to remission if treated. In face of such a need, clinical diagnosis remains difficult. At the (very) early stage, inflammatory arthritis often has an atypical presentation with progression towards RA that can vary in speed. Autoantibodies (RF and ACPA) are useful in RA diagnosis as recently recognised by their inclusion in the new diagnostic EULAR 2010 criteria. However, they both lack sensitivity in early disease (<50%) [213] even if ACPA specificity is quite high (over 95%) [214].

The ideal RA diagnostic biomarker should therefore be characterised by high specificity and sensitivity, both close to 100%. An ideal biomarker should also detect the presence of RA at early stages. Few, if any, biomarker testing systems achieve these levels of sensitivity and specificity although this can be approached by improvement of the assays. In advanced disease (i.e., fully developed RA), biological differences between healthy and disease states are easily detected. In contrast, in early disease, the biological distinctions between healthy and disease states or alternative diagnosis are often more subtle, and clear differentiation even for a gold standard becomes more challenging. Therefore, the evaluation of a candidate diagnostic biomarker requires an infallible diagnosis to be established which in RA remains difficult [215].

Cytokines and other soluble factors are prime candidates for diagnostic biomarkers. Several studies investigated their expression using variable methods (ELISA, multiplex assays, or gene expression) and material (tissue and body fluids). However, few studies actually compared very early inflammatory arthritis with differential outcome and still use healthy individuals or established disease patients as controls. Cytokines detected in joints were not different in 12- month disease duration compared to more advanced RA [216]; however, these findings remain to be established in very early disease. Even if right and left RA knee showed similar profiles (IL-6, IL-8, IL-10, and IFN-gamma, high expression of IL-1beta, TNF-alpha, and TGF-beta, low levels of IL-2 and GM-CSF, and no detectable IL-4 or IL-5) [217], the same pattern was observed in other diseases such as seronegative spondyloarthropathy or OA with different levels of expression.

Using Luminex technology with the blocking of heterophilic antibody, increased levels of TNF-alpha, IL-1beta, IL-6, IL-12P40, IL-13, and several chemokines (CXCL10, CCL11, CCL2, and IL-8) were observed in sera from RA patients with <6-month symptom duration compared to HC [23]. The profile was specific to RA and not reproduced in established AS or SpA but was not investigated in patients with early inflammatory symptoms who did not progress towards RA. The profile was also restricted to ACPA-positive patients suggesting increased inflammation associated with autoreactivity. In addition, ACPA was closely related to RF in this study (titres were directly correlated), questioning the efficiency of the RF-blocking methodology used as most cytokine levels were also related to ACPA levels.

In a similar study [158] comparing already diagnosed RA patients of less than 6-month symptom duration with established AS and PsA, a multiplex biomarker platform (combining cytokines, bone turnover markers, metalloproteinases, inflammatory markers, and several citrullinated epitopes) established a signature again including cytokines such as TNF-alpha, IL-1alpha and beta, IL-6, IL-12p40, IL-15, IL-17, GM-CSF, and eotaxin. However, most were also present in AS and PsA (TNF-alpha, IL-1beta, IL-6, IL-17, and eotaxin) and others were associated with autoantibody positive disease (IL-1alpha, IL-12p70, and IL-15).

Studies truly investigating early diseases and the value of cytokines as diagnostic biomarkers in a predictive manner are few. SF from early inflammatory arthritis patients before diagnosis established that patients with persistent symptoms on development of RA showed increase in Th2 cytokines (IL-4 and IL-13) but not Th1 (IFN-gamma) [218]. IL-17 was also increased however only in established RA [218]. In individuals who donated serum samples and later developed RA, a multiplex study showed significant increased levels of cytokines related to T cell activation (IL-2, IL-6), inflammation (IL-1beta, IL-1RA, and TNF-alpha), Th1 (IL-12 and IFN-gamma), Th2 (IL-4, IL-13, and eotaxin), and immune regulation (IL-10), while chemokines, stromal cell-derived cytokines, and angiogenic-related markers were elevated in patients after the development of RA rather than in individuals before the onset of RA [219]. Levels were particularly increased in ACPA-positive and RF-positive individuals. However, in all three studies, every cytokine and chemokine tested were increased (even if not significantly) and again particularly in ACPA/RF-positive patients, whereas other studies demonstrated reduction (i.e., IL-2 and IL-7). Therefore, technical issues related to heterophilic antibody interference may have to be considered when interpreting these data. A similar preclinical RA study [220] showed no detectable cytokine more than 5 years before RA onset, but during the 5-year interval before diagnosis, increased levels were associated with an increased likelihood of the risk of developing RA (IL-1 alpha, IL-1beta, IL-1RA, IL-4, IL-10, TNF-alpha, and soluble TNF-RI).

In established RA as well as in patients with less than 24-month symptom duration, reduced levels of circulating IL-7 have been reported [177, 196]. IL-7 is a pleiotropic cytokine regulating peripheral T cell homeostasis, notably in RA [177, 221, 222]. However, IL-7 is highly expressed in the joints of RA patients [196, 197, 223, 224], and such discrepancies between low systemic levels and high expression at disease site have also been reported in systemic sclerosis [225] and recently in ulcerative colitis and Crohn's disease [226, 227]. A cohort of 250 sera from patients with very early symptoms suggesting a possible evolution towards RA (less than 6-month duration and 5-year follow-up) designed to discover diagnostic biomarkers demonstrated the potential of IL-7 as a biomarker [2].

### 3.4. Cytokines as Markers for Treatment Selection and Response to Therapy

Biological therapies (cytokine blockade or receptor antagonism) nowadays appear very effective in chronic inflammatory conditions such as RA, however, in a limited number of patients, with up to 40% nonresponse. Considering the cost of such therapies, biomarker prediction response and allowing for selection of the most appropriate biological treatment would have considerable impact. Most authorities recommend starting therapy with biologics after the failure to respond to at least one disease-modifying agent in RA. However, due to the limited number of studies, there is little guidance about which biological agent to select although anti-TNF remains the most commonly used.

RA patients not responding to anti-TNF showed higher synovial fluid IL-6 at baseline amongst elevated levels of IL-1beta, IL-1RA, IL-2, IL-4, IL-8, IL-10, IL-17, IFN-gamma, G-CSF, GM-CSF, and TNF-alpha. In contrast, responders had elevated IL-2 and G-CSF. In plasma, however, levels were not significantly predicting response, and IL-6 levels decreased posttreatment. In this study, SF cytokine clustering revealed 6 groups of patients with possibly underlying different cellular pathologies, and IL-6, IL-2, and G-CSF in SF may be useful in predicting response to anti-TNF [228]. Recently, we also showed that serum IL-6 was significantly higher at baseline in rituximab nonresponders and that a significant reduction followed treatment in responders only despite adequate B cell depletion in nonresponders [229]. Multivariate logistic regression analysis of synovial cytokine expression showed that TNF at baseline could only explain *∼*10–15% of the variance in response to TNF blockade [230], suggesting that TNF expression itself would have a limited role in relation to personalised health care. Synovial tissue analysis associated absence of sign of improvement with increased TNF and MMP-3 expression [231, 232]. In contrast, another study showed response to be associated with higher TNF bioactivity in the blood [233], which is more convenient for personalised medicine.

To date, several studies using blood have used gene expression rather than ELISA. CCL4, IL-8, and IL-1beta discriminated between responders and nonresponders to anti-TNF [234]. Several gene signatures have been published so far (some including IL-8, IL-2R) [235–238] with a sensitivity of 90% and a specificity of 70% [237] and 94.4% sensitivity and 85.7% specificity for the response to anti-TNF treatment [238]. Response to anti-TNF (etanercept) was associated with reduced levels of IL-6 and increased IL-23 and IL-32 posttreatment while there was no change in nonresponders; however, no baseline level had predictive value [239].

Recently, several interferon signalling related signatures have emerged as potential biomarkers of response to biological therapies [240–242] as well as for the progression of “at risk” individuals to symptomatic arthritis [243]. Such signatures are interesting as they most likely reflect an immunological status that is favourable to responding or not to therapy, although they are not really linked to the presence/absence of interferon. Indeed, these signatures combined different sets of intracellular signalling factors and transcriptional regulators (between 8 and 15 markers) and are measured through gene expression (using mostly qPCR).

## 4. Conclusion

Assays measuring known diagnostic biomarkers are commonly used in clinical practice. In fact, it has been reported that about 70% of the decisions made by physicians are based on the results provided by those tests [244]. However, the implementation of novel biomarkers into clinical practice proves to be a long and challenging process, which includes convincing physicians. The assessment of the impact of using the biomarker on general health is an essential step to guarantee the uptake of the biomarker into clinical practice and to further optimise its use. This area of research is likely to become increasingly important as more biomarkers enter clinical practice [245]. Given the complexity and heterogeneous nature of RA, it is unlikely that a single cytokine may provide sufficient discrimination. Many reliable cytokine assays are nowadays available with multiplex formats taking the lead (although this may not be an appropriate solution in RA due to RF interferences). These have established clinical utility for other diseases and purposes and should be easily (technically) transferable to rheumatology, although the exact performance characterization and quality assurance for the specific cytokines of interest in RA may need to be established. At present, limitation in RA lies more in the disease related complexity of networks, the elucidation of the respective role, and the redundant effect that one cytokine may have with another.

Finally, multiple biomarker signatures potentially using genetic as well as proteomic markers may represent a more realistic approach for the future of personalised medicine in RA. Such multifactorial analysis may potentially reveal patterns rather than individual biomarkers. As such, it is interesting that IL-7 alone was able to predict diagnostic at very early disease stage, whereas more complex combination of markers may be needed to predict response to therapy and define subsets of patients with more advanced and heterogonous disease.

## Figures and Tables

**Table 1:** Summary of reported preanalytical precautions to be enforced to measure some of the main cytokines. A large amount of the literature is contradictory, most likely due to different analytical discrepancies in the evaluation of the effect of preanalytical conditions. This table aims to provide a review of the literature available; however, there is no thoroughly enough conducted study allowing us to suggest definitive guidance as to the best condition to process samples universally (i.e., allowing for any cytokines to be tested). Furthermore, the inflammatory nature of RA further complicates such issues.

	Serum or plasma	Delays in separation (whole blood pending processing)	Storage condition (after separation)	Sensitivity to freeze-thawing (F/T) cycles
IL-1 (alpha and beta)	(i) Both are used [26] (ii) Higher heparin plasma concentrations compared to serum [27] (iii) Higher levels in EDTA plasma than in heparin plasma [28]	(i) Increased levels with delays in processing when kept at RT (ii) No significant change for up to 4 days of delayed processing in samples from healthy people; however, there is significant decrease in samples from trauma patients [29] (iii) Significant increase in serum, after delay of 48 h at 4°C, with RA patients, but in plasma there is an increase only if kept at RT [30] (iv) Prolonged delays before separation result in increased endotoxin-induced cytokine release in contaminated tubes [31, 32]	(i) Storage at 4°C results in an increase (ii) Heparin plasma showed time-dependent increases in concentration [31]	No significant change in stability in plasma/serum for up to 6 F/T cycles [26]

IL-2	(i) Heparin plasma concentrations are higher than in serum [27] (ii) Comparable or higher levels in EDTA plasma compared to heparin [28]	No significant change for up to 4 days of delayed processing in samples from healthy people; however, there is a significant decrease when processing samples for trauma patients [29]		

IL-4	(i) Heparin plasma concentrations are higher than in serum [27] (ii) Higher levels of IL-4 in EDTA plasma than in heparin [28] and higher concentration in serum than in heparin plasma [33]	(i) No significant change for up to 4 days of delayed processing [29] (ii) No significant change for serum or EDTA plasma stored before centrifugation at 4°C, RT, and 35°C [30]		

IL-5	Slightly higher levels in EDTA plasma than in serum [30]	(i) No significant change for up to 4 days of delayed processing [29] (ii) Plasma levels significantly increased if separation delayed by 4 h stored at 4°C. Further increase if stored at RT [30] (iii) Serum levels increased with delayed processing for 24 h at 4°C or 4 h at RT [30]		

IL-6	(i) Serum and EDTA plasma samples are comparable while levels in heparin and citrate plasma are lower [34] (ii) Serum levels are higher than EDTA plasma [30] (iii) Serum and EDTA, citrate, or heparin plasma gave comparable results [28, 35] (iv) Heparin plasma levels are higher than those of serum [27] and this anticoagulant is not recommended due to *ex vivo* Il-6 release prior to assay [31] (v) Endotoxin contamination (LPS) triggers release in heparin compared to EDTA plasma [32, 36]	(i) Reduced levels when samples are left unseparated for 24 h at 4°C or RT [26] or 4 h at RT [34] (ii) Significant reduction in stability and recovery with time at RT [26] (iii) Increased levels with delays in processing when left at RT (iv) No change in samples stored at 4°C for 24 h before centrifugation [35] (v) No change when left at 4°C or 20°C for up to 4 days before centrifugation [37] (vi) No significant change for up to 4 days of delayed processing in samples from healthy people; however, there is a significant decrease when processing samples from trauma patients [29] (vii) Plasma levels unchanged when stored for up to 3 h at 37°C but afterwards, an increase is observed [31] (viii) Increased endotoxin-induced cytokine release in contaminated tubes with delays in processing [32]	No change in levels in serum stored at 4°C, −20°C, and −30°C [37]	(i) No significant change for up to 6 F/T cycles (ii) No significanceobserved after 2, 3, and 4 times of repeated F/T cycles [37] (iii) No significant effect for up to 3 F/T cycles in EDTA plasma and serum but inconsistent stability in heparin plasma [26, 34]

IL-7	(i) No significant difference between plasma and serum IL-7 levels [38] (ii) Serum levels are significantly higher than in plasma [33] (iii) Heparin plasma concentrations are higher than in serum [27]	(i) 2 to 4 hours of delayed processing decrease IL-7 plasma levels [38] (ii) With 2 to 4 hours of delayed processing, serum levels are stable [38] (iii) No significant change for up to 4 days of delayed processing [29]		Stable for up to 3 F/T cycles

IL-8	(i) Comparable levels in heparin plasma and in serum [27] (ii) Higher serum levels than in heparin plasma [33] (iii) Lower levels in EDTA plasma than in heparin [28] (iv) LPS induced release in whole blood is up to 100 times higher in heparin versus EDTA plasma [36]	(i) Increased levels with delays in processing if left at RT [29] (ii) Stable levels if stored at 4°C		

IL-9		No significant change for up to 4 days of delayed processing [29]		

IL-10	(i) Higher levels in serum than in plasma [39] (ii) Lower levels in EDTA plasma than in heparin [28]	(i) Increased levels with delays in processing if left at RT (ii) The longer the delay, the less stable the levels (iii) No significant change for up to 4 days of delayed processing in samples from healthy people; however, there was a significant decrease in samples from trauma patients [29]	Storage temperature affects stability: the higher the temperature, the faster the decline [37]	No significant decline in levels observed after 2, 3, or 4 times of repeated F/T cycles [37]

IL-12 (p70 & p40)	Heparin and EDTA plasma levels are higher than in serum [27, 28, 30, 33]	(i) Levels decrease with delayed processing [29] (ii) No significant change for up to 4 days of delayed processing [29] (iii) Increase in serum after 48 h of delayed processing at 4°C and 4 h at RT [30] (iv) Stable in plasma for over 48 h at 4°C and for up to 48 h at RT [30]		

IL-13	(i) Heparin plasma levels are higher than those of serum [27] (ii) Comparable levels in EDTA and heparin plasma [28]	No significant change for up to 4 days of delayed processing [29]		

IL-15		No significant change for up to 4 days of delayed processing [29]		

IL-16				Decrease after the 5th F/T cycle [40]

IL-17	(i) Lower levels in EDTA plasma than in heparin [28, 33] (ii) Higher levels in serum than in any plasma (EDTA, citrate, and heparin) [33] (iii) Higher levels in EDTA plasma than in serum [30]	(i) No significant change for up to 4 days of delayed processing in samples from healthy people; however, there was a significant decrease in samples from trauma patients [29] (ii) Plasma levels increased if separation delayed by 4 h at 4°C with further increase with time (up to 24 h) [30]		

IL-18	Similar levels in serum and EDTA plasma [30]	No changes in EDTA levels over 48 h at 4°C, and significant increase after 24 h at RT [30]		

TNF-alpha	(i) Comparable results in serum and EDTA plasma [39] (ii) Lower levels in sodium citrate plasma (iii) Higher heparin and EDTA plasma levels than in serum [26, 27, 30] (iv) LPS induced release of TNF-alpha 20 times higher when in heparin compared to EDTA plasma [36] (v) Endotoxin induces high release [32, 41]	Contradictory data: (i) Reduced levels with delays in processing when kept at 4°C and RT [26, 42] (ii) Increased levels with delays in processing if left at RT [34, 43] (iii) No significant change for up to 4 days of delayed processing [29] (iv) Time-dependent increases in levels with delays at 37°C in heparin plasma [31]	(i) Reduction in samples kept at RT for 20 days (ii) Relatively stable in samples stored at 4°C [39] (iii) Stable at −70°Cfor over 9 months [42]	Contradicting data: (i) Levels increased with successive F/T cycles [26, 34] (ii) No differences reported in plasma and serum for up to 10 F/T cycles [39]

TGF-beta 1	(i) Higher levels in serum than plasma (citrate, EDTA) due to platelet degranulation during the clotting process [30, 44–46] (ii) EDTA plasma is not recommended because of the extreme interindividual variation of PLT activation and concurrent *in vitro* GF release [44] (iii) Sodium citrate can be used but is not as effective or reliable [44] (iv) CTAD (citrate theophylline dipyridamole adenosine) is recommended as it blocks the *in vitro* release of growth factors from PLTs (v) Plasma concentrations should be corrected by simultaneous measurement of markers of platelet degranulation [47]	(i) Increased levels with delay when plasma is left at RT or 37°C [48] due to platelet degranulation and release [45] (ii) Lower level in serum if left at 4°C than at RT [49] (iii) Speed of centrifugation affects recovery in plasma (2,500 ×g for 30 min yields lower levels than 1,200 ×g for 10 min) [49]		<5% deviation from baseline value in serum upon successive F/T cycles (for up to 100 F/T cycles) [48]

sCD40-ligand	(i) Use of platelet poor/free plasma is recommended as it is [50] higher in serum than in plasma (EDTA, citrate, and heparin) due to clot retraction and sCD40L shedding from the platelet surface [33, 50] (ii) EDTA anticoagulated plasma samples are not appropriate for sCD40L measurements [51]	(i) Increased levels after 3 h of delay in processing [50] (ii) Serum levels increase with time in delayed processing [50] (iii) No significant changes in serum or plasma levels detected after storage at 4°C for up to 48 h (iv) Significant loss observed in serum and plasma, left at RT [52] (v) Decreased levels with increasing centrifugation *g* values (200–13 000 g), which gradually deplete plasma of platelets [52]	(i) Loss in serum and plasma kept for over 4 h at RT [40] (ii) No change while stored at 4°C (iii) Significant decrease after 24 h at 37°C [40]	(i) Stable for up to 3 F/T cycles [52] (ii) Increased after 5 or 10 F/T cycles [40]

IFN-gamma	(i) Collection in sterile pyrogen free tubes is very essential (ii) Serum levels are higher than in plasma (EDTA, citrate, and heparin) [33] (iii) Heparin plasma levels are higher than in serum [27] (iv) Levels in heparin plasma are higher than in EDTA plasma [33] (v) Levels in EDTA plasma are higher than in heparin plasma [28]	(i) Significant reduction with time at both 4°C and RT in serum and EDTA tubes [26] (ii) IFN-gamma decreases if processing is delayed [29]		Stable for up to six F/T cycles [26]

**Table 2:** Description and characteristics of assays measuring cytokines.

Cytokine assay technique	Description	Characteristics
Bioassays	*Bioassays* (commonly used shorthand for *biological assays*) are typically assays by which the potency or the nature of a substance is estimated by studying its effects on living organisms They can be conducted to measure the concentration/effects of a cytokine on a living cell Example: IL-2 bioassay using an IL-2 dependent cell line that will undergo apoptosis in the absence of IL-2 in a dose dependent manner They require tissue culture facility	Low specificity Semiquantitative detection Highly sensitive with detection limit < 1 pg/mL Narrow analytical range Time consuming (24–96 h) Low precision (CV = 20–100%) Drug interference Laborious protocol with high staff cost

ELISA	Quantitative detection of a molecule (bioactive and inactive) based on its capture by an antibody followed by its detection by another antibody coupled with a detection (commonly named ELISA) It requires specialised equipment	Less sensitive than bioassays <10 pg/mL Relatively large sample volume Wide analytical range High reagent cost Excellent precision (CV = 5–10%) No drug interference Simple and relative rapid protocol

Solid phase assay (Luminex)	Technology based on the detection of dyed microbeads capturing a cytokine with a first antibody and quantifying it with a second antibody coupled with fluorescence and lasers detection It allows multiplex detection	Small sample volume Lower sensitivity than ELISA Large range of analytes Sensitive to interferences from heterophilic antibodies (i.e., naturally occurring anti-antibodies), anti-cytokine antibodies, and presence of soluble receptors

Other solid phase assays	Mosaic ELISA ELISA like technology allowing multiple detection of cytokines in a 96-well plate format by spotting capture antibodies	Small sample volume Lower sensitivity than ELISA Only 8 analytes per test

Molecular techniques	All techniques allowing mRNA quantification Earlier detection of cytokines at transcriptional level however may not represent cytokine production and release They require specialised equipment	Highly specific Highly sensitive as they can detect changes at the single-cell level Complete analytical range (from single cytokine to as many as needed) Excellent precision No drug interference Simple and relative rapid protocol Relatively high cost
